# Evaluation of the Adequacy of General Anesthesia in Cesarean Section by Bispectral Index

**Published:** 2013-09

**Authors:** Sayed Mohammad Reza Hadavi, Elaheh Allahyary, Saman Asadi

**Affiliations:** Anesthesiology and Critical Care Research Center, Department of Anesthesiology, Faghihi Hospital, Shiraz University of Medical Sciences, Shiraz, Iran

**Keywords:** Bispectral index, Awareness, Recall, Cesarean section

## Abstract

**Background: **Awareness and recall, though not common, are the major hazards of general anesthesia, especially in Cesarean section (C/S) because of the absence of benzodiazepine and opioids for a significant time during anesthesia. In this study, the Bispectral Index (BIS), end-tidal isoflurane, and hemodynamic parameters were examined to evaluate the depth of the routine general anesthetic technique in C/S.

**Methods: **This study was carried out on 60 parturient patients undergoing elective C/S. A standardized anesthetic technique was applied: induction with Thiopental (4-5 mg/kg) and Succinylcholine (1.5-2 mg/kg) as well as maintenance with O2, N2O, and isoflurane. Electrocardiogram, heart rate, blood pressure, Spo2, end-tidal isoflurane concentration, BIS, and any clinical signs of inadequate depth of anesthesia such as movement, sweating, lacrimation, coughing, and jerking were continuously monitored and recorded at 16 fixed time points during anesthesia.

**Results:** A median BIS of less than 70 (range: 42-68) was obtained on all occasions during surgery; however, at each milestone, at least 20% of the patients had BIS values above 60. Hemodynamic parameters increased significantly in some patients, especially during laryngoscopy and intubation. No patient experienced recall or awareness.

**Conclusion: **The currently used general anesthetic technique in our center appears inadequate in some milestones to reliably produce BIS values less than 60, which are associated with lower risk of awareness. Therefore, with respect to such desirable outcomes as good Apgar and clinical status in neonates, we would recommend the application of this method (if confirmed by further studies) through larger dosages of anesthetic agents.

## Introduction

Adequate anesthesia to prevent pain, awareness, and recall is the major role of the anesthesiologist. This is achieved by a balanced administration of analgesic, hypnotic, and amnesic drugs. Some different methods are used to evaluate the depth of anesthesia during different types of surgeries; these include spontaneous surface electromyogram (SEMG), lower esophageal contractility (LOC), heart rate variability (HRV), and electroencephalogram and its derived indices.^1^^,^^2^

Cesarean section (C/S) renders parturient patients at risk of inadequate anesthesia because of rapid sequence induction, avoidance of opioids and Benzodiazepine until the delivery of the newborn, and limited volatile concentration.^3^^,^^4^ In a study in 2004, the risk of inadequate depth of anesthesia in C/S with Sevoflurane was 20- 45%.^4^ Therefore, the light plane of general anesthesia for the fetal safety during C/S may give rise to post-traumatic stress disorder.^5^^, ^^6^ It is clear that the prevention of inadequate depth of anesthesia is a very important goal and as such merits further research.

The routine approach for evaluating the depth of anesthesia is the assessment of hemodynamic parameters and subjective signs such as movement, sweating, and lacrimation, which are not adequately sensitive and specific.^7^ Since 1977, several studies have sought to determine whether Bispectral Index (BIS) monitoring is a reliable tool for the analysis of the anesthetic depth.^8^ An FDA-approved method, the BIS is adequately sensitive for the evaluation of the depth of anesthesia and is believed to be useful for the detection of light anesthesia by processing the patient’s electroencephalogram (EEG).^9^^,^^10^ Accordingly, the BIS can be drawn upon to prevent anesthetic complications such as awareness, recall, and unintentional hemodynamic changes – not least in some types of operations with significant risk of inadequate depth of anesthesia like C/S. The BIS ranges from 0 (EEG silence) to 100 (fully awake and alert).^10^ A BIS range of 40 to 60 denotes an adequate level of anesthesia.^11^ In short, the BIS presents an evaluation of the depth of anesthesia in surgical patients.^12^ It should also be noted that titrating anesthetic agents via BIS monitoring can decrease the total dose of hypnotic drugs mandatory for an acceptable depth of anesthesia.^10^

The purpose of the present study was to evaluate BIS monitoring in C/S and its relevance to hemodynamic parameters,subjective signs of light anesthesia, awareness, recall, and end-tidal volatile concentration in 60 parturient patients.

## Participants and Methods

After obtaining approval from the Institution’s Ethics Committee and provision of written informed consent by all the patients, 60 parturient patients (the American Society of Anesthesiologists [ASA] physical status I-II) scheduled for elective lower-segment C/S under general anesthesia were enrolled in the study. Population selection was carried out after a review of relative articles and according to statistical analysis. The exclusion criteria included a history of mental disease and anticipated difficult intubation. 

After at least 3-5 minutes of preoxygenation in a 10-15° tilted position, anesthesia was induced by 4-5 mg/kg Sodium Thiopental and 1.5-2 mg/kg Suxamethonium. After the neonatal delivery, Midazolam (0.03 mg/kg), Fentanyl (1.5 micg/kg), Morphine (0.1 mg/kg), and Atracurium (0.4 mg/kg after the return of spontaneous respiration) were given intravenously. Anesthesia was maintained by O2, N2O, and isoflurane (1-1.5% before delivery and 0.5-1% subsequently).

Electrocardiogram (ECG), blood pressure (BP), HR, SpO2, temperature, and BIS were continuously monitored as were end-tidal isoflurane, N2O, and CO2 concentrations using a calibrated multiple gas analyzer (Varmus or Dragger ) during the anesthesia. The patients received Fentanyl (1 µg/kg) intravenously if there were any clinical signs in favor of inadequate depth of anesthesia including an increase by more than 20% of the pre-anesthetic values in HR and mean arterial blood pressure (MAP), lacrimation, coughing, sweating, and movement. All the data were recorded by one person, unaware of anesthetic management. Also, the anesthetist was blinded to the BIS values. The BIS, HR, and BP were measured and recorded at 16 designated points of sequential events during anesthesia: before induction; 30 seconds after laryngoscopy; intubation; skin incision; retraction of abdominal rectus muscles; uterine incision; fetal delivery; uterine curettage; uterine closure; abdominal lavage; closure of peritoneum; closure of subcutaneous tissue; shutoff of isoflurane; skin closure; reversal administration; and eye opening. isoflurane and N2O were stopped upon the start and completion of skin closure, respectively. Reversal of muscle relaxation (by Neostigmine and Atropine) was performed during skin closure. The patients were asked to open their eyes at one-minute intervals for extubation. The time period from cessation of inhalational agents to eye opening was noted. All the patients were interviewed at the time of discharge from post-anesthesia care unit and 12-24 hours after that for determination of awareness or recall.

*Questions*

-Could you alert anyone during surgery? 

-Did you have any recall while surgery was being done?

-Do you have any dream about your surgery or operating room?

*Statistical Analysis*

This study is a descriptive analysis of the correlation between the BIS and changes in the end-tidal isoflurane concentration. 

The statistical analyses were performed through SPSS software. The association between the BIS and changes in the end-tidal isoflurane concentration, HR, and MAP was assessed using data from all the perioperative time points. 

## Results

**Figure 1:**
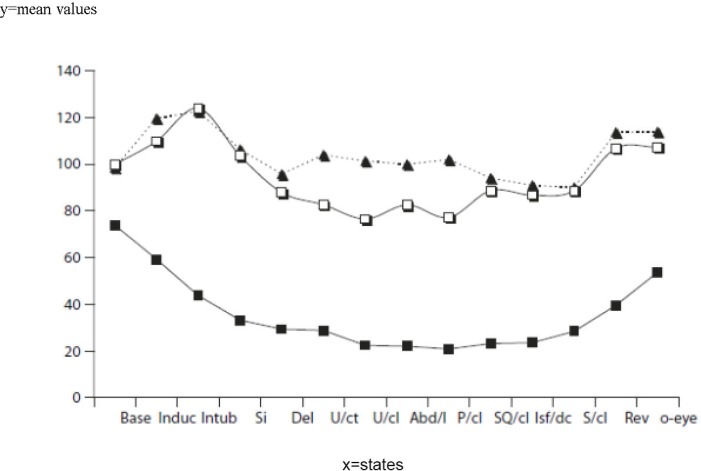
The mean values of mean arterial blood pressure (MAP) (white squares) and heart rate (HR) (black triangles) at predetermined time points at designated events along with the measurement of the Bispectral Index (BIS) (black squares) are depicted herein. Base: Baseline; Induc: After induction; Intub: Intubation; Si: Skin incision; Del: Delivery; U/ct: Uterine curettage; U/cl: Uterine closure; Abd/l: Abdominal lavage; P/cl: Peritoneal closure; SQ/cl: Subcutaneous closure; Isf/dc: Cessation of isoflurane; S/cl: Skin closure; Rev: Reversal administration; O-eye: Eye opening

**Figure 2:**
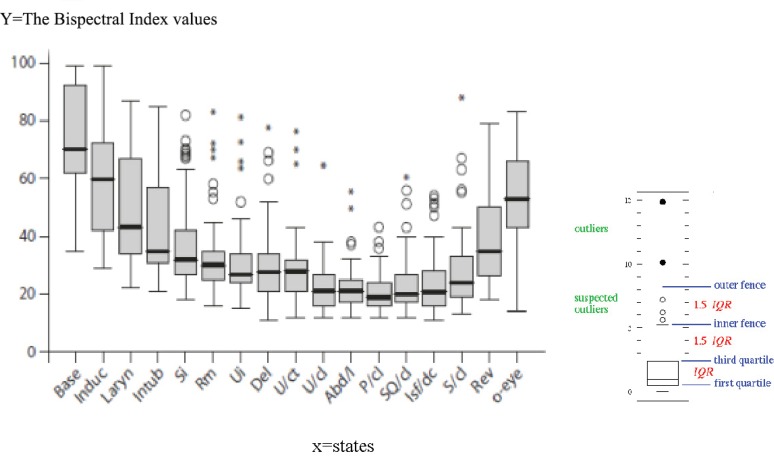
The Bispectral Index (BIS) values (25, 50, and 75 percentile) at predetermined time points are illustrated herein. Base: Baseline; Induc: After induction; Laryn: Laryngoscopy; Intub: Intubation; Si: Skin incision; Rm: Retraction of rectus muscles; Del: Delivery; U/ct: Uterine curettage; U/cl: Uterine closure; Abd/l: Abdominal lavage; P/cl: Peritoneal closure; SQ/cl: Subcutaneous closure; Isf/dc: Cessation of isoflurane; S/cl: Skin closure; Rev: Reversal administration; O-eye: Eye opening; Box: First quartile, median, third quartile; Box whiskers: The lowest datum still within 1.5 IQR of the lower quartile, and the highest datum still within 1.5 IQR of the upper quartile, IQR=3rd quartile-1st quartile; ○: Datum within 1.5-3 IQR of the upper quartile (between inner and outer fences; *Datum outer than 3 IQR of the upper quartile (above outer fence

All the 60 enrolled parturients (17-39 years old) completed the study and all the collected data are presented (table 1 and figures 1-2). The newborns’ Apgar scores at 1 and 5 minutes were 8±0.7 (6-9) and 9±0.6 (7-10), respectively. Due to the very short time intervals defined for the purposes of this study, MAP could not be measured at some designated points such as laryngoscopy and uterine incision in some patients.

**Table 1:** Duration of the various phases of the anesthetic and surgical events

**Anesthetic and surgical phases**	**Duration (min)** **mean±SD (min-max)**
Anesthesia course	58±87 (49-71)
Operation course	39±14 (31-73)
Induction to delivery	6.1±2.1 (3.3-11.2)
Uterine incision to delivery	4.4±0.5 (1.6-5.3)
Reversal administration to eye opening	6.3±1.7(2-14)

There was no patient with significant uterine atony or postpartum hemorrhage (attributable to isoflurane concentration). No patient reported intraoperative awareness or recall at the postoperative interview. Clinical signs of inadequate depth of anesthesia occurred in 28 (46%) patients at least at one point. Of these 28 patients, 13 (21%) had lacrimation, sweating, or sialorrhea but 15 (25%) had body movement (extremities, facial muscles, and tongue) during laryngoscopy and intubation, coughing, or bucking, which necessitated the anesthetist’s intervention. The number and percentage of the patients with BIS values more than 60 (with a high probability of awareness) at each milestone are shown in table 2. As is shown in figure 1, MAP and HR were increased at intubation; this was associated with an initial decrease (during induction) and subsequent increase in the BIS values. The BIS, MAP, and HR thereafter changed in the same manner (downward until cessation of anesthetic with an upward trend after that). The BIS values (25, 50, and 75 percentile) at the predetermined time points are presented as a Box Plot (figure 2).

**Table 2:** Number and percentage of the patients with a bispectral index above 60

**Time points**	**Number of cases**	**Percentage**
Baseline	60	100
Induction	13	21.7
Laryngoscopy	10	16.7
Intubation	24	40
Skin incision	47	78.3
Retraction of rectus muscles	32	53.3
Delivery	24	40
Uterine curettage	19	31.7
Uterine closure	14	23.3
Abdominal lavage	9	11.5
Peritoneal closure	12	20
Subcutaneous closure	14	23.3
Cessation of isoflurane	13	21.7
Skin closure	39	65
Reversal administration	53	89.7
Eye opening	59	98.3

## Discussion

In spite of the remarkable trend toward the use of regional anesthesia in Western Europe and USA, many C/S patients still undergo the operation under general anesthesia elsewhere in the world.^13^^-^^16^ Some of these patients are frightened of the prospect of a needle in their back or wakefulness during major abdominal surgery.^16^ Regional anesthesia is liable to be very problematic, unfeasible, or contraindicated in some other patients with special medical problems.^17^^-^^25^

The rates of awareness and occurrence of unpleasant dreams during general anesthesia for C/S in different studies have been reported between 0.13-7% and 17%, respectively. These figures are clearly higher in other cases, probably leading to severe psychological sequelae.^26^^,^^27^ The use of clinical signs is not of much help to the anesthetist in precisely assessing the level of hypnosis through general anesthesia. However, the use of relevant variables such as unresponsiveness, HR, BP, and the anesthetic method has proven useful enough to explain the causes of awareness.^28^^,^^29^ Clinical signs to infer the depth of anesthesia, which rely on changes in the autonomic nervous system, are obscured by concurrent drugs such as adrenergic blockers, coexisting diseases such as hypertension, and side effects of agents given during surgery such as tachycardia associated with isoflurane. Moreover, hypovolemia, hypoxia, hypercapnia, or inadequate analgesia rather than inadequate anesthesia may lead to such manifestations. Because many purposeful movements are due to reflexes at the level of the spinal cord, they cannot essentially denote that the patient is awake.^30^ BIS monitoring provides an EEG–derivative index, a numerical array from 1 to 100 that has been reported to correlate with the central nervous system (CNS)-depressant effects of anesthetic drugs. This monitoring tool is sensitive to the hypnotic effects of inhaled anesthetics as well as Propofol in a dose-dependent manner. It is also deserving of note that the BIS is not sensitive enough for the assessment of analgesia and evaluation of the effect of opioids during general anesthesia.^19^^,^^31^^-^^33^

Sodium thiopental requirement is decreased during pregnancy.^34^ The present study was started with 4 mg/kg Sodium Thiopental, but the administration of this dose to the second patient was accompanied by aggressive movements of all the extremities and severe bucking with intubation. Therefore, 5 mg/kg of Sodium Thiopental was preferred for the remaining 58 patients afterward. 

According to the results of the present study, the course of general anesthesia during C/S can be divided into three different phases according to changes in the BIS values and homodynamic parameters:

*A- Induction to Intubation*

The initial increases in BP and HR observed at laryngoscopy and intubation in spite of the downward trend in the BIS values most likely reflected autonomic or brainstem responses to noxious stimuli rather than a sign of inadequate depth of anesthesia. The absence of opiates at induction time in C/S was associated with a significant sympathetic response and hemodynamic changes with painful stimuli. The BIS values showed significant decreases, with the median value changing from 98 before induction to 49, 42, and 45.5 after induction (BIS<60 is considered acceptable depth of anesthesia), laryngoscopy, and intubation, respectively (figure 1).

*B- Intubation to Uterine Closure*

The BIS values had a downward trend after an initial increase at skin incision, which was correlated with the same trend in the hemodynamic parameters (due to decrease in painful stimuli).

*C- Uterine Closure to the End of Anesthesia*

The BIS values and hemodynamic parameters had the same trend with an upward direction. The increase in the BIS values was predictable after decreasing isoflurane from 1.5% to 0.75% at the time of neonatal delivery with a short delay (until uterine closure), which was needed for the decrease in plasma and brain tissue isoflurane concentration. The rise in hemodynamic parameters can be explained from two points of view: 

1- It could have been secondary to the gradual decrease in the depth of anesthesia due to the drop in isoflurane concentration.

2- After a significant bleeding due to uterine incision and placental delivery, gradual replacement of intravascular volume might have led to hemodynamic stability and increases in previously decreased BP. 

*Assessment of Clinical Signs of Awareness during Anesthesia*

Clinical signs of awareness were seen in 46% of the patients at least at one time point during anesthesia. Of them, 21% were in the forms of lacrimation, sweating, or sialorrhea and 25% in the forms of movements (extremities, facial muscles, and tongue) or bucking during laryngoscopy and intubation. 

Like hemodynamic changes, findings such as lacrimation, salivation, and sweating can be explained as neuroendocrine responses to noxious stimuli rather than the clinical signs of inadequate depth of anesthesia, but any different body movements should probably be considered as the clinical signs of inadequate depth of anesthesia (with or without inadequate muscle relaxation), which was seen in 15 patients. 

The most frequent time points for the clinical signs of inadequate anesthesia were intubation (23%) and skin incision (17%), while these signs were not seen in more than 5% of the patients at each of the other time points. This is reasonable because the physiologic stress of intubation and skin incision is the strongest stress during the course of surgery and anesthesia. 

*Recall of Events of Anesthetic Period*

None of these 60 patients remembered the events from their anesthesia course during the first 24 hours after surgery. 

*Adequacy of the Depth of Anesthesia According to Bispectral Index*

The results of this study revealed:

1- In all the 60 patients, most of the studied milestones were not associated with an acceptable BIS score as an index for adequate depth of anesthesia. 

2- Except for 3 milestones (laryngoscopy, uterine closure, and peritoneal lavage), at all the other time points, at least 20% of the patients had non–acceptable BIS indices. 

3- The most frequent times for inadequate depth of anesthesia (BIS>60) were skin incision, skin closure, and retraction of rectus muscles (53%). 

It is clear that laryngoscopy and intubation were the most painful procedures in the course of anesthesia and surgery; nonetheless, the relatively acceptable BIS scores in these stages could be due to the very short interval between the induction of anesthesia and laryngoscopy of the patients. Another probability is that the BIS score was not a real-time monitoring for the depth of anesthesia.

It is obvious that whether we consider the BIS values as the best index for the assessment of the adequacy of anesthesia depth, clinical signs of awareness, or hemodynamic parameters, we should accept that at some time points during general anesthesia for C/S such as intubation, skin incision, and retraction of rectus muscles, there is no acceptable depth of anesthesia in a significant number of patients with the routine present regimen of anesthesia. It is notable that maximum doses of Sodium Thiopental (5 mg/kg), isoflurane (1.5%), and Scholine (2 mg/kg) were used in all the 60 patients. On the other hand, some studies have revealed that pregnant women have lower values of the BIS than non-pregnant ones (after similar doses of drugs in early pregnancy), which may be the case in late pregnancy as well.^34^

A review of the neonatal Apgar scores and maternal hemodynamic parameters revealed that none of our patients had evidence of drug overdosage in spite of receiving maximum programmed dosages of the used drugs. The results of this study suggest that dosages more than those currently in use may be appropriate for the induction and maintenance of general anesthesia in C/S. In this regard, it should be noted that: 

1- In another study with doses higher than usual (5-7 mg/kg Pentothal and end-tidal isoflurane of 1% instead of 0.5% in similar studies), no significant neonatal depression or maternal hemodynamic derangement was seen.^3^ Nevertheless, further researches with more precise neonatal evaluations, especially neurobehavioral scales, are needed.

2- All of the limited number of studies conducted hitherto have recruited pregnant women with ASA=I or II. An important question is, therefore, whether or not the BIS and hemodynamic values similar to those seen in this study are acceptable in pregnant women with cardiovascular disease. For example, at intubation, 25% of the patients had BIS>68, systolic BP>168 mm Hg, diastolic BP>102 mm Hg, and HR>126 beats per minute and 5% of the patients had BIS≥77, systolic BP>186 mm Hg, diastolic BP>132 mm Hg, and HR>140 beats per minute. 

The BIS is thought to be a good monitor for the assessment of the depth of anesthesia and the titration of anesthetic drugs on this basis. A BIS less than 60 correlates with adequate anesthesia, 60-70 with light anesthesia (or deep sedation), and more than 70 with the possibility of recall. However, we did not have recall in spite of BIS values more than 70 in some patients, and this was also seen in similar previous studies.^3^^-^^5^

We should also remember that: 

1- The above-mentioned BIS classification is a good index for the assessment of recall, but not an absolutely reliable index for the assessment of awareness, and factors such as delay in spontaneous memory and retrograde amnesia due to post-delivery administration of Midazolam and opioids can exert some influence. 

2- The rest of traumatic memory can act as a psychological stimulant and lead to post-traumatic stress disorder.

3- BIS values of 73 or more have been observed to co-exist with some degrees of explicit memory in the absence of conscious recall.

In a study in 2005, the BIS was mentioned as a good monitoring tool for the evaluation of the depth of anesthesia and a median BIS of 60 was introduced as being adequate during C/S.^27^ Another study, aside from recommending the BIS as a reliable monitoring way in the course of surgery, stated that the maintenance of anesthesia during C/S with isoflurane or Propofol was acceptable in terms of sufficient hypnosis.^9^ In a study in 2012, Thiopental and Propofol were compared using the BIS as an acceptable marker of the depth of anesthesia vis-à-vis the induction of hypnosis in C/S.^35^ In that study, Propofol was found to be as effective as Thiopental for the initiation of anesthesia. 

Several studies have revealed that adding N2O to anesthetic regimens has no influence on BIS values in spite of producing a smoother course of anesthesia. As a result, it is probable that N2O had some contribution toward the decrease in the rate of awareness and recall in our study without influence on the BIS values. Also, awareness and recall were less than that estimated on the basis of the BIS values. 

## Conclusion

Overall, on the basis of the results of this study and review of previous studies, we believe that more frequent and larger studies are needed to evaluate the BIS as an ideal monitoring tool for anesthetic depth and to recommend it as a means of ensuring the absence of awareness and recall. 

Of course, as the results of this study reveal, the current methods of general anesthesia for C/S cannot ensure a desirable depth of anesthesia.
